# Factors Contributing to Educational Failure Among Secondary School Students in Erbil City

**DOI:** 10.7759/cureus.66953

**Published:** 2024-08-15

**Authors:** Salih Ahmed Abdulla

**Affiliations:** 1 Department of Community Nursing, College of Nursing, Hawler Medical University, Erbil, IRQ

**Keywords:** erbil city, secondary school students, academic performance, contributing factors, educational failure

## Abstract

Background and aim

Educational failure among secondary school students in Erbil City is influenced by a complex interplay of factors. This study is crucial as it aims to conduct an in-depth investigation into the various factors contributing to educational failure among secondary school students in Erbil City. Specifically, the study seeks to explore how students' attitudes toward school and learning influence their academic performance, including their levels of engagement, motivation, and perceptions of school. Additionally, it aims to examine the impact of teacher-related factors, such as teaching quality, classroom management, and teacher-student relationships, on students' academic success. Furthermore, the study will assess the role of school resources, including the availability of educational materials and facilities, and the influence of extracurricular activities on students' overall academic outcomes.

Methods

This cross-sectional study was conducted from March 15, 2023, to June 5, 2024, in nine secondary high schools in Erbil City. A total of 280 students participated in the study, using purposive sampling. The questionnaire comprised demographic information and a self-structured questionnaire with 30 items assessing attitude factors, school teacher factors, and school potential and extracurricular activity factors. Statistical analysis was performed using IBM SPSS Statistics for Windows, Version 28 (Released 2021; IBM Corp., Armonk, New York, United States), with frequency and percentage used for qualitative variables and mean and standard deviation for quantitative variables.

Results

A total of 280 students participated in the study. The overall mean scores were 1.57 ± 0.37 for school potential and extracurricular activity factors, 1.42 ± 0.43 for attitude factors, and 1.18 ± 0.35 for school teacher factors, indicating that school potential and extracurricular activity factors had the most significant impact on academic performance. Among these, support for students with special needs (262 (93.6%)), strict internal school rules (250 (89.3%)), and availability of career counseling services (239 (85.4%)) were reported as major factors. Attitude factors such as attending school being a pleasure (224 (80.0%)) and seeking assistance from teachers (217 (77.5%)) were significant, while school teacher factors had a lesser impact, with maintaining order in classrooms (252 (90.0%)) and marking absent students' names (262 (93.6%)) being reported as minor factors.

Conclusions

The study showed that school potential and extracurricular activities were the main causes of educational failure, followed by attitude factors. School teacher factors had the least impact. Policymakers and healthcare providers should prioritize targeted interventions to improve school infrastructure, support students with special needs, and enhance career counseling services. Furthermore, efforts should be made to promote positive attitudes toward school and learning and to bolster teacher support and training to better address educational challenges.

## Introduction

Educational failure, defined as the inability of students to meet expected academic standards, resulting in poor performance, grade repetition, or dropout, is a growing concern worldwide with far-reaching consequences for individuals, families, and society as a whole [1,2]. In Erbil City, Iraq, the issue is particularly pressing, with a significant number of students struggling to meet academic standards and complete their secondary education [3]. However, there is a lack of comprehensive data on the risk of educational failure both globally and specifically in Erbil City. This gap in the data underscores the novelty and importance of this study, as it aims to shed light on the crucial roles of socioeconomic status, family support, and school environment in educational outcomes. This alarming trend highlights the urgent need for a comprehensive understanding of the factors contributing to educational failure and the development of targeted interventions to support at-risk students. Addressing this issue is essential not only for the well-being and future prospects of individual students but also for the overall development and progress of Erbil City and its communities.

Numerous variables have been identified as potential contributors to educational failure among secondary school students in Erbil City. These factors can be broadly categorized into individual, family, and school-related domains [4,5]. At the individual level, students' motivation, self-efficacy, and academic skills have been shown to significantly impact their educational outcomes [6]. Family factors, such as parental education, involvement, and socioeconomic status, also play a crucial role in shaping students' academic trajectories [7,8]. Moreover, school-related factors, including teacher quality, classroom environment, and access to resources, have been linked to students' educational success or failure [9,10]. Understanding the complex interplay between these variables is essential for developing effective strategies to support students at risk of educational failure in Erbil City.

The relationship between these variables and educational failure is complex and multifaceted. For instance, students from low-income families may face additional challenges, such as limited access to educational resources and increased stress, which can negatively impact their academic performance [11]. Similarly, students attending schools with inadequate facilities and under-qualified teachers may struggle to engage with the curriculum and develop the necessary skills for academic success [12]. Existing research on the topic has provided valuable insights into the interplay between these factors and their influence on educational outcomes. However, there is a need for more contextualized studies that take into account the unique social, cultural, and economic conditions of Erbil City.

Furthermore, the impact of educational failure extends beyond the individual student, affecting families, communities, and the broader society [13]. Students who struggle academically are more likely to experience low self-esteem, decreased motivation, and limited future prospects, which can lead to a cycle of disadvantage and marginalization [14,15]. Moreover, educational failure can have long-term economic consequences, as students who do not complete their secondary education may face difficulties in securing stable employment and contributing to the development of their communities [16]. Addressing the factors contributing to educational failure in Erbil City is, therefore, not only a matter of individual well-being but also a critical step toward promoting social equity, economic growth, and sustainable development.

One of the most impactful barriers that students experiencing educational failure face is stigma. This stigma, whether from peers, teachers, or even family members, can exacerbate feelings of shame and inadequacy, further hindering their academic performance [17]. In this context, cognitive behavioral therapy (CBT) emerges as a powerful intervention. While CBT is widely used to treat conditions such as suicide, depression, and anxiety [18,19], it can also support students facing educational failure. CBT helps students challenge and reframe negative thought patterns, empowering them to develop healthier coping strategies and improve their academic engagement [20]. By addressing the psychological impacts of stigma and providing tools for emotional resilience, CBT can play a crucial role in helping students overcome barriers to academic success.

Despite the growing body of research on educational failure, significant gaps remain in our understanding of the specific factors contributing to this issue in Erbil City. While previous studies have identified various individual, family, and school-related variables associated with educational failure, many factors have not been thoroughly examined within the context of Erbil City's educational system. There is a need to extend the existing literature by exploring these overlooked factors and investigating their interactions. By addressing these gaps, researchers can provide valuable insights and recommendations for policymakers, educators, and families seeking to support at-risk students and promote educational success in Erbil City. Therefore, this study aimed to investigate the factors contributing to educational failure among secondary school students in Erbil City.

## Materials and methods

Study design, setting, and period

This cross-sectional study was conducted in Erbil City and involved nine secondary high schools: Cheeman High School, Zheen High School, Laban High School, Halala High School, Vanas High School, Shorsh High School, Kurdistan High School, Azadi High School, and Rizgari High School. These schools were selected using purposive sampling to ensure a diverse and representative sample of the student population across Erbil City. The selection was influenced by several factors, including geographic distribution, school size, and the demographic diversity of the student body. Importantly, most schools approached for the study declined to participate, and these nine schools were the ones that gave consent to be included in the research. Data were collected from March 15th, 2023, to June 5th, 2024.

Sample size

The sample size was determined using the infinite population sample size formula. We considered a 95% confidence interval, a margin of error of 5.86%, a population proportion of 50%, and a Z score of 1.96. The final sample size was 280 students. The final sample size was calculated according to the formula below:

$n=(z^2&times;p ̂(1-p ̂))/&epsilon;^2$

Where Z is the z score, ϵ is the margin of error, N is the population size, and p^ is the population proportion

Inclusion/exclusion

The inclusion criteria required students to have experienced academic failure at least once and those who agreed to participate in the study. The exclusion criteria included students who did not complete at least 90% of the questionnaire, those who were not currently enrolled in a secondary school, and those who had transferred to a different school within the last six months.

Study tools and data collection

The questionnaire consisted of two parts. The first part collected demographic data, including age, gender, grade level, working status, household task assistance, school difficulties, mathematics and science grades, desired educational level, parents' education level, student-parent relationship, and family income. The second part contained a self-structured questionnaire with 30 items assessing attitude factors, school teacher factors, and school potential and extracurricular activity factors. To ensure accuracy, the questions were translated from English to Kurdish using a forward-backward method, and the translation was verified by specialists in the field. Data were collected by distributing questionnaires to participants who met the inclusion criteria. Students were given 15-20 minutes to complete the questionnaire.

Pilot study

The questionnaire was tested in an initial study with 30 students from January 5th to February 5th, 2023, to assess the internal consistency and reliability of the items before they were used in the actual study. The internal consistency of the items was calculated using Cronbach's alpha [21]. The overall Cronbach's alpha was 0.88, indicating very good reliability. To ensure content validity, the questionnaire was reviewed by 10 nursing professors from various specialties. The data from this initial study were excluded from the final analysis.

Measures

Sociodemographic Characteristics

The demographic variables included age, gender, grade level, working status, household task assistance, school difficulties, mathematics and science grades, desired educational level, parents' education level, student-parent relationship, and family income.

Self-structured questionnaire

The second part of the questionnaire was a self-structured instrument containing 30 items divided into three sections: attitude factors, school teacher factors, and school potential and extracurricular activity factors, with 10 items each. Students responded with "yes" or "no" to each item. For scoring, "no" responses were assigned 2 points, indicating major factors, while "yes" responses were assigned 1 point, indicating minor factors. The scoring was based on the overall mean of each factor and each item, with the maximum score being 2 and the minimum score being 1. The internal consistency of the questionnaire was assessed using Cronbach's alpha [21], yielding a coefficient of 0.88, indicating very good reliability.

Ethical approval and informed consent

This study adhered to the guidelines set by the Institutional Research Ethics Board and the Declaration of Helsinki. Ethical approval was granted by Hawler Medical University, College of Nursing on March 7th, 2023, with the code number 30. For students who were under 18 years old, a formal consent form was sent to their parents for signature. Only after receiving consent from both parents and students were these students eligible to participate in the study. Students who were 18 years or older provided their own informed consent by signing the consent form.

Statistical analysis

Data were summarized and reported using frequency and percentage for qualitative variables. Quantitative variables with a normal distribution were presented as mean and standard deviation. Data analysis was performed using IBM SPSS Statistics for Windows, Version 28 (Released 2021; IBM Corp., Armonk, New York, United States), with higher means indicating more significant factors.

## Results

Demographic characteristics

A total of 280 students participated in the study. The majority (122 (43.6%)) were 17 years old. The gender distribution showed that 157 (56.1%) were female, and 123 (43.9%) were male. Most of the students were in stages 11 and 12 (120 (42.9%) each), while 40 (14.3%) were in stage 10. Regarding working status, 140 (50.0%) of the students worked, but not every day, 120 (42.9%) did not work, and 20 (7.1%) worked every day. More than half of the students (150 (53.6%)) never assisted with household tasks. The majority (250 (89.3%)) reported having no difficulties in school, and 168 (60.0%) had marks above 50% in Mathematics and Sciences. Most students (231 (82.5%)) desired to be high school graduates. Fathers' and mothers' education levels were predominantly at the elementary school level (201 (71.8%) and 182 (65.0%), respectively). The relationship between students and parents was satisfactory for 182 (65.0%) of the participants, and 234 (83.6%) reported intermediate family earnings. Detailed demographic characteristics are presented in Table 1.

**Table 1:** Demographic Characteristics of the Students and Their Caregivers N: Number; %: Percentage; SD: Standard Deviation

Items	Categories (n=280)	N	%
Age (year)	15	29	10.4
	16	35	12.5
	17	122	43.6
	18	94	33.6
Gender	Male	123	43.9
	Female	157	56.1
Stages	10	40	14.3
	11	120	42.9
	12	120	42.9
Working status	No	120	42.9
	Yes, but not every day	140	50.0
	Yes, every day	20	7.1
Assisting with household tasks	Never	150	53.6
	About 1 hour a day	80	28.6
	About 2 hours a day	30	10.7
	More than 2 hours a day	20	7.1
Do you have any difficulties in school?	Yes	30	10.7
	No	250	89.3
Marks in mathematics and sciences	Less than 50 percentage	112	40.0
	More than 50 percentage	168	60.0
The desired educational level for the student	Going to leave school	35	12.5
	High school graduate	231	82.5
	College Graduate	14	5.0
Father's level of education	Illiterate	7	2.5
	Elementary school level	201	71.8
	High school level	42	15.0
	Institutional level	20	7.1
	University level	10	3.6
Mother's level of education	Illiterate	14	5.0
	Elementary school level	182	65.0
	High school level	21	7.5
	Institutional level	42	15.0
	University level	21	7.5
Relationship between student and parents	Excellent	30	10.7
	Well-being	57	20.4
	Satisfactory	182	65.0
	Inadequate	11	3.9
Family earning	High	18	6.4
	Intermediate	234	83.6
	Low	28	10.0

Positive attitudes toward school and learning factors

The study examined factors related to students' attitudes toward school and learning. The overall mean score for these factors was 1.42 ± 0.43. Some factors were identified as major, indicating their negative impact on students' academic performance. Following classroom rules and completing assignments were considered minor factors by 273 (97.5%) of the students, suggesting that most students adhere to these expectations and it contributes to their educational success. Similarly, only 182 (65.0%) of the students reported finding homework enjoyable, indicating that the majority do not view it as a hindrance to learning. However, 224 (80.0%) of the students considered attending school to be a major factor, suggesting that a significant proportion do not enjoy it, which can affect their academic performance. Seeking assistance from teachers when facing learning problems was also reported as a major factor by 217 (77.5%) of the students, emphasizing the need for improved teacher support to address educational challenges. Table 2 provides a comprehensive overview of these findings.

**Table 2:** Assessment of Positive Attitudes Toward School and Learning Factors Contributing to Educational Failure Among Secondary School Students in Erbil City N: 280 represents the total number of participants in the study; SD: Standard Deviation.

Attitude factors	Reporting item as a minor factor N (%)	Reporting item as a major factor N (%)	Total (N)	Mean (SD)
Attending school is always a pleasure	56 (20.0%)	224 (80.0%)	280	1.80 (0.40)
Completing homework is enjoyable	182 (65.0%)	98 (35.0%)	280	1.35 (0.48)
In the classroom, I follow the rules and complete assignments	273 (97.5%)	7 (2.5%)	280	1.03 (0.20)
When facing learning problems, I seek assistance from my teacher	63 (22.5%)	217 (77.5%)	280	1.78 (0.42)
I occasionally choose to skip classes that I believe are unimportant	170 (60.7%)	110 (39.3%)	280	1.39 (0.49)
I frequently arrive late to school	189 (67.5%)	91 (32.5%)	280	1.33 (0.47)
I participate actively in class discussions	205 (73.2%)	75 (26.8%)	280	1.27 (0.44)
I feel motivated to achieve good grades	133 (47.5%)	147 (52.5%)	280	1.53 (0.50)
I have a positive relationship with my classmates	208 (74.3%)	72 (25.7%)	280	1.26 (0.44)
I enjoy participating in school clubs and activities	152 (54.3%)	128 (45.7%)	280	1.46 (0.50)
Overall Mean Score	-	-	280	1.42 (0.43)

School teacher factors

The study also assessed school teacher factors contributing to educational failure. The overall mean score for these factors was 1.18 ± 0.35. Most teacher-related factors were reported as a minor by the majority of the students, indicating that these aspects have a lesser negative impact on student's educational outcomes. Maintaining order in classrooms was reported as a minor factor by 252 (90.0%) of the students, marking absent students' names by 262 (93.6%), and having positive relationships with students by 264 (94.3%). However, teachers providing respectable and effective teachings and additional help outside of class hours were reported as major factors by 103 (36.8%) of the students, implying that there is room for improvement in the quality of teaching and extra support provided by teachers. Further details can be found in Table 3.

**Table 3:** Assessment of School Teacher Factors Contributing to Educational Failure Among Secondary School Students in Erbil City N: 280 represents the total number of participants in the study; SD: Standard Deviation.

School teacher factors	Reporting item as a minor factor N (%)	Reporting item as a major factor N (%)	Total (N)	Mean (SD)
The instructors at my school try their best to maintain order in classrooms	252 (90.0%)	28 (10.0%)	280	1.10 (0.30)
The names of absent students are routinely marked by the teachers	262 (93.6%)	18 (6.4%)	280	1.06 (0.25)
Our school's teachers provide respectable and effective teachings	177 (63.2%)	103 (36.8%)	280	1.37 (0.48)
I'm confident that the lessons being taught to us have been well-planned	259 (92.5%)	21 (7.5%)	280	1.08 (0.26)
In general, teachers urge us to achieve high academic standing	259 (92.5%)	21 (7.5%)	280	1.08 (0.26)
The relationships between students and instructors are generally positive	264 (94.3%)	16 (5.7%)	280	1.06 (0.23)
Teachers provide additional help outside of class hours	177 (63.2%)	103 (36.8%)	280	1.37 (0.48)
Teachers use a variety of teaching methods	226 (80.7%)	54 (19.3%)	280	1.19 (0.39)
Teachers provide timely feedback on assignments	193 (68.9%)	87 (31.1%)	280	1.31 (0.46)
Teachers encourage student participation in class	227 (81.1%)	53 (18.9%)	280	1.19 (0.39)
Overall Mean Score	-	-	280	1.18 (0.35)

School potential and extracurricular activity factors

The study assessed school potential and extracurricular activity factors contributing to educational failure. The mean score for these factors was 1.57 ± 0.37. Several factors were reported as major by the majority of students, indicating their significant negative influence on academic performance. Support for students with special needs was reported as a major factor by 262 (93.6%) of the students, suggesting a lack of adequate support. Strict internal school rules were reported as a major factor by 250 (89.3%) of the students. The availability of career counseling services was reported as a major factor by 239 (85.4%) of the students. The organization of sporting events, trips, and visits by the school, as well as the availability of extracurricular clubs and societies, were also reported as major factors by 217 (77.5%) and 220 (78.6%) of the students, respectively. This suggests that a lack of engaging extracurricular activities may negatively impact students' overall educational experience. Opportunities for student leadership roles were reported as a major factor by 185 (66.1%) of the students, indicating that more chances for student involvement in decision-making processes could be beneficial. Table 4 provides a detailed presentation of these findings.

**Table 4:** Assessment of School Potential and Extracurricular Activity Factors Contributing to Educational Failure Among Secondary School Students in Erbil City N: 280 represents the total number of participants in the study; SD: Standard Deviation.

School potential and extracurricular activity factors	Reporting item as a minor factor N (%)	Reporting item as a major factor N (%)	Total (N)	Mean (SD)
The school organizes sporting events, trips, and visits	63 (22.5%)	217 (77.5%)	280	1.78 (0.42)
Availability of libraries and multimedia rooms at school	218 (77.9%)	62 (22.1%)	280	1.22 (0.41)
The school is located in a secure neighborhood	270 (96.4%)	10 (3.6%)	280	1.04 (0.19)
Internal school rules are strict	30 (10.7%)	250 (89.3%)	280	1.89 (0.31)
Class sizes are large	194 (69.3%)	86 (30.7%)	280	1.31 (0.46)
Presence of technical instructional aids in classrooms	207 (73.9%)	73 (26.1%)	280	1.26 (0.44)
Availability of extracurricular clubs and societies	60 (21.4%)	220 (78.6%)	280	1.79 (0.41)
Opportunities for student leadership roles	95 (33.9%)	185 (66.1%)	280	1.66 (0.47)
Availability of career counseling services	41 (14.6%)	239 (85.4%)	280	1.85 (0.35)
Support for students with special needs	18 (6.4%)	262 (93.6%)	280	1.94 (0.25)
Overall mean score	-	-	280	1.57 (0.37)

## Discussion

The present study aimed to investigate the factors contributing to educational failure among secondary school students in Erbil City, focusing on attitude factors, school teacher factors, and school potential and extracurricular activity factors. Overall, the results indicated that school potential and extracurricular activity factors were the most significant in affecting students' academic performance, followed by attitude factors, while school teacher factors were found to be the least significant.

Educational failure among secondary school students is a complex issue that can have far-reaching consequences for individuals and society as a whole. In Erbil City, the specific challenges faced by students and the education system are unique, making it essential to understand the factors that contribute to academic underperformance. Despite the importance of this issue, there is a lack of comprehensive data on the specific factors influencing educational failure in this region. Given the significance of these details, this study aimed to investigate the role of attitude factors, school teacher factors, and school potential and extracurricular activity factors in shaping the academic outcomes of secondary school students in Erbil City.

The demographic profile of the study participants, featuring a slightly higher number of female students, and a majority in the later stages of secondary education, closely mirrors the typical student population in Erbil City. The significant proportion of students who work, albeit not every day, aligns with findings from other studies that have examined the impact of student employment on academic performance [22,23]. Additionally, the predominance of parents with elementary school education levels in this study is consistent with research conducted in developing countries, where parental education is a significant predictor of students' academic success [24,25]. These demographic insights underscore the broader socio-economic context influencing the academic experiences of secondary school students in this region.

The study's findings regarding positive attitudes toward school and learning factors are consistent with previous research that emphasizes the importance of a supportive and engaging learning environment [26]. The majority of students in this study reported adhering to classroom rules and completing assignments, which is similar to findings from other studies that have linked positive student behaviors to academic success [27,28]. However, the high proportion of students who do not find attending school to be a pleasure and the need for improved teacher support when facing learning problems are unique challenges identified in this study, highlighting the specific areas for improvement in the context of Erbil City secondary schools.

Interestingly, school teacher factors were found to be the least significant in contributing to educational failure among the factors studied, which contrasts with some previous research that has emphasized the critical role of teacher quality and support in shaping academic outcomes [29,30]. While most students in this study reported positive aspects of teacher performance, such as maintaining order in classrooms and having positive relationships with students, the identified areas for improvement, such as the quality of teaching and the availability of additional support outside of class hours, are consistent with findings from other studies [31,32].

The most significant factors contributing to educational failure, as revealed by the study, were school potential and extracurricular activity factors. The lack of adequate support for students with special needs, strict internal school rules, and the absence of career counseling services were identified as major factors by a substantial majority of the students. These findings align with previous research that has highlighted the importance of inclusive education practices, flexible school policies, and comprehensive guidance services in promoting academic success [33-36]. However, the limited availability of engaging extracurricular activities, such as sporting events, trips, visits, clubs, and societies, is a unique finding of this study, emphasizing the need for a greater focus on providing diverse opportunities for student engagement and development in Erbil City secondary schools.

While this study provides valuable insights into the factors contributing to educational failure among secondary school students in Erbil City, it also demonstrates several strengths. The study's robust sample size and comprehensive demographic analysis offer a detailed understanding of the student population. Additionally, the identification of key factors such as parental education and student employment provides a solid foundation for future interventions. Future research could benefit from employing longitudinal designs and incorporating objective measures of academic performance to further validate and extend these findings. Moreover, exploring the effectiveness of targeted interventions addressing the identified factors could provide practical recommendations for improving educational outcomes in Erbil City secondary schools. These efforts could lead to the development of evidence-based strategies that enhance the academic success of secondary school students in this region.

Limitations of the study

A key limitation of the study is that its findings are specific to the nine secondary high schools in Erbil City and may not be generalizable to other regions or populations. Additionally, the use of purposive sampling, while practical for this study's specific needs, may introduce selection bias, which could impact the overall validity of the results. Furthermore, the fact that some schools refused to participate in the study may have affected the sample size and potentially influenced the representativeness of the findings.

## Conclusions

The study identified school potential and extracurricular activities as the most significant contributors to educational failure, followed by attitude-related factors, with school teacher factors being the least significant. To address these issues, policymakers and healthcare providers should prioritize enhancing school infrastructure, providing robust support for students with special needs, and improving career counseling services. Additionally, fostering positive attitudes toward school and learning is crucial. Strengthening teacher support and training to address educational challenges better is also essential. Future research should investigate the specific barriers within these factors and evaluate the effectiveness of targeted interventions. Longitudinal studies are recommended to assess the long-term impact of these interventions on students' academic performance.

## Appendices

Title: factors contributing to educational failure among secondary school students in Erbil City

Questionnaire

This questionnaire is integral to our article titled " Factors Contributing to Educational Failure Among Secondary School Students in Erbil City" Your participation is entirely voluntary and highly valued. The questionnaire guarantees complete anonymity; hence, please do not provide any personal identifiers such as your name. Your insights are important to this study, and I am grateful for the time you dedicated to completing this survey.

Code …………..

Name of the school………………………………

**Table 5:** Socio-demographic Characteristics Questionnaire Respondents are asked to tick the option that fits them for each category

Part 1: Sociodemographic Characteristics	Options	Response
X1: Age	15	
	16	
	17	
	18	
X2: Gender	Male	
	Female	
X3: Stages	10	
	11	
	12	
X4: Working status	No	
	Yes, but not every day	
	Yes, every day	
X5: Assisting with Household Tasks	Never	
	About 1 hour a day	
	About 2 hours a day	
	More than 2 hours a day	
X6: Do You Have Any Difficulties in School	Yes	
	No	
X7: Marks in Mathematics and Sciences	Less than 50 percentage	
	More than 50 percentage	
X8: The Desired Educational Level for the Student	Going to leave school	
	High school graduate	
	College Graduate	
X9: Father's Level of Education	Illiterate	
	Elementary school level	
	High school level	
	Institutional level	
	University level	
X10: Mother's Level of Education	Illiterate	
	Elementary school level	
	High school level	
	Institutional level	
	University level	
X11: Relationship Between Student and Parents	Excellent	
	Well-being	
	Satisfactory	
	Inadequate	
X12: Family Earning	High	
	Intermediate	
	Low	

Part two: factors contributing to educational failure questionnaire

Please read each statement and circle a number 0, 1, which indicates how much the statement applies to you. There are no right or wrong answers. Do not spend too much time on any statement.

**Table 6:** Factors Contributing to Educational Failure Questionnaire Respondents are asked to rate each statement based on how much it applied to them. The rating scale is as follows: 0: No, 1: Yes, No: Number.

Item No.	Statement	No	Yes
1.	Attending school is always a pleasure	0	1
2.	Completing homework is enjoyable	0	1
3.	In the classroom, I follow the rules and complete assignments	0	1
4.	When facing learning problems, I seek assistance from my teacher	0	1
5.	I occasionally choose to skip classes that I believe are unimportant	0	1
6.	I frequently arrive late to school	0	1
7.	I participate actively in class discussions	0	1
8.	I feel motivated to achieve good grades	0	1
9.	I have a positive relationship with my classmates	0	1
10.	I enjoy participating in school clubs and activities	0	1
11.	The instructors at my school try their best to maintain order in classrooms	0	1
12.	The names of absent students are routinely marked by the teachers	0	1
13.	Our school's teachers provide respectable and effective teachings	0	1
14.	I'm confident that the lessons being taught to us have been well-planned	0	1
15.	In general, teachers urge us to achieve high academic standing	0	1
16.	The relationships between students and instructors are generally positive	0	1
17.	Teachers provide additional help outside of class hours	0	1
18.	Teachers use a variety of teaching methods	0	1
19.	Teachers provide timely feedback on assignments	0	1
20.	Teachers encourage student participation in class	0	1
21.	The school organizes sporting events, trips, and visits	0	1
22.	Availability of libraries and multimedia rooms at school	0	1
23.	The school is located in a secure neighborhood	0	1
24.	Internal school rules are strict	0	1
25.	Class sizes are large	0	1
26.	Presence of technical instructional aids in classrooms	0	1
27.	Availability of extracurricular clubs and societies	0	1
28.	Opportunities for student leadership roles	0	1
29.	Availability of career counseling services	0	1
30.	Support for students with special needs	0	1
